# A Systemic Review on the Diagnostic Accuracy of Point-of-Care Ultrasound in Patients With Undifferentiated Shock in the Emergency Department

**DOI:** 10.7759/cureus.23188

**Published:** 2022-03-15

**Authors:** Ingvar Berg, Kris Walpot, Hein Lamprecht, Maxime Valois, Jean-François Lanctôt, Nadim Srour, Crispijn van den Brand

**Affiliations:** 1 Emergency Medicine Department, Haaglanden Medical Centre, The Hague, NLD; 2 Emergency Medicine Department, University Hospital Leuven, Leuven, BEL; 3 Division of Emergency Medicine, Stellenbosch University, Cape Town, ZAF; 4 Emergency Medicine Department, Hôpital Charleslemoyne, Montreal, CAN; 5 Respiratory Medicine Department, Hôpital Charleslemoyne, Montreal, CAN; 6 Emergency Medicine Department, Erasmus Medical Center, Rotterdam, NLD

**Keywords:** resuscitation, pocus, ultrasound, hypotension, shock

## Abstract

Early identification of the shock type and correct diagnosis is associated with better outcomes. Previous studies have suggested that point-of-care ultrasound (POCUS) increases the diagnostic accuracy of patients in undifferentiated shock. However, a complete overview of the diagnostic accuracy of POCUS and the related treatment changes when compared to standard care is still limited. Our objective was to compare POCUS against standard practice regarding the diagnostic accuracy and specific therapeutic management changes (fluid volume administration and vasopressor use) in patients with undifferentiated shock in the emergency department (ED).

We conducted a systematic review in concordance with the Preferred Reporting Items for Systematic Reviews and Meta-Analyses. A systematic search was performed using Embase, PubMed, Cochrane Central Register for Controlled Trials, and clinicaltrials.gov. Two physicians independently selected the articles and assessed the quality of the studies independently with the Quadas-2 tool. All included studies used POCUS in adult patients in undifferentiated shock and described diagnostic accuracy or specific therapeutic management changes (fluid volume administration or vasopressor use) and compared this to standard care. The primary outcome was diagnostic accuracy. Secondary outcomes were the amount of fluid administered and vasopressor use in the ED. Only articles published after 1996 were included.

There were 10,805 articles found of which 6 articles were included. Four out of six studies reported diagnostic accuracy, three reported on fluid administration and vasopressors. We found that the diagnostic accuracy improved through the use of POCUS when compared to the standard care group, increasing overall diagnostic accuracy from 45-60% to 80-89% when combined with clinical information. There was no significant difference in fluid administration or vasopressor use between the groups.

In our systematic review, we found that the use of POCUS in patients that presented with undifferentiated shock in the ED improved the diagnostic accuracy of the shock type and final diagnosis. POCUS resulted in no changes in fluid administration or vasopressor use when compared to standard care. However, the results should be interpreted within the limitations of some of the studies that were included in the review.

## Introduction and background

Shock represents 0.4% to 1.3% of all emergency department (ED) presentations and up to one-third of all intensive care unit (ICU) admissions [1-3]. It is associated with high morbidity and in-hospital mortality of up to 48% [1,4-6]. Early recognition by the use of shock alerting systems has been shown to decrease mortality. Therefore, it seems reasonable to assume that rapid and accurate detection of the cause of shock has the potential to improve patient outcomes further [7].

While physical examination alone is unreliable to accurately determine the correct cause of hypotension [8,9], evidence suggests that point-of-care ultrasound (POCUS) has the potential to obtain good diagnostic accuracy in patients with hypotension in the ED [10,11]. The use of POCUS has gained widespread acceptance in recent years and is progressively becoming the standard of care in the evaluation of critically ill patients [12]. Pneumothorax, pericardial tamponade, fluid hypovolemia, left ventricular failure, and right ventricular strain can all be detected by POCUS [13,14]. As a result, many approaches to optimize and organize the use of POCUS in shock have been described [4,15-22]. However, there is a lack of overview of the diagnostic accuracy of POCUS in undifferentiated shock patients that present to the ED. 

Therefore, the objective of this systematic review is to compare POCUS against standard practice regarding the diagnostic accuracy and specific therapeutic management changes (fluid volume administration and vasopressor use) in patients with undifferentiated shock in the ED.

## Review

Materials and methods

Literature Review

The reporting of the present review followed the Preferred Reporting Items for Systematic Reviews and Meta-Analyses (PRISMA) statement [23].

Search Strategy

A search strategy was constructed with medical subject headings and keywords focusing on "POCUS," "shock," and "emergency department" (Appendices: Table 4). An initial search was conducted on September 14, 2015, and a follow-up search was conducted on November 27, 2020. The following databases were searched: EMBASE through OVID (January 1, 1996 to November 27, 2020), PubMed (January 1, 1996 to November 27, 2020), and Cochrane Central Register for Controlled Trials (May 24, 2019). We searched the reference lists of appropriate studies, most relevant guidelines and consulted the clinicaltrials.gov registry (April 12, 2020), after which we contacted the authors of all ongoing trials on this topic for preliminary results.

Inclusion and Exclusion Criteria

We included studies that used POCUS in adult patients in undifferentiated shock, described diagnostic accuracy or specific therapeutic management changes (fluid volume administration or vasopressor use), and compared this to standard care. The following studies were excluded: (i) studies conducted outside of an ED setting, (ii) differentiated shock (e.g. trauma, septic shock), (iii) studies that included pregnant patients or patients <18 years old, (iv) studies that did not use ultrasound as a diagnostic tool to determine or exclude the cause of shock in a clinical setting, (v) studies that examined trans-esophageal ultrasound, and (vi) studies lacking a control group where ultrasound was (initially) not performed.

Data Collection and Processing

**Figure 1:**
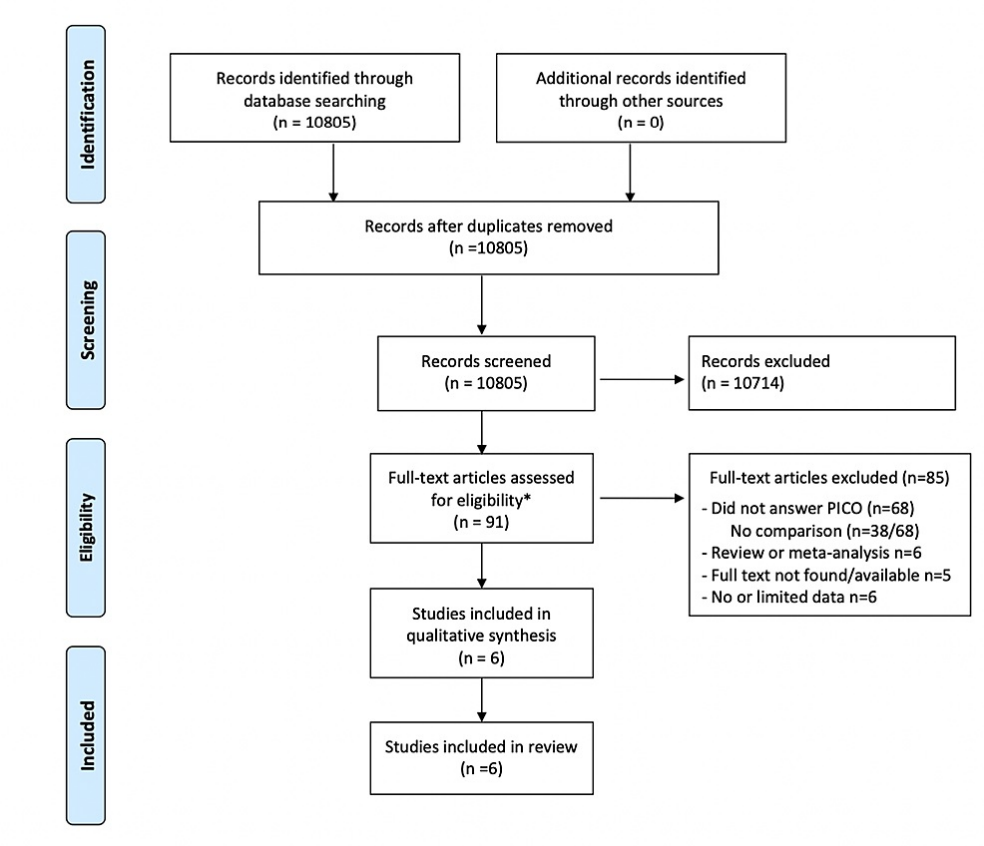
Preferred Reporting Items for Systematic Reviews and Meta-analyses (PRISMA) flow diagram. PICO: Population, Intervention, Comparison, Outcomes of study as mentioned in methods section [23].

The primary outcome was the difference in diagnostic accuracy between the POCUS and standard-care groups. The diagnostic accuracy was defined as the percentage of occurrences of the correct diagnosis with or without POCUS. The correct diagnosis was defined as the gold standard that was used in the article (e.g., final diagnosis at discharge). Secondary outcomes were differences in IV fluid administration (total mL of fluids administered during the ED stay) and vasopressor use in the ED between the POCUS and standard care groups. For studies to be eligible, data related to at least one of these outcomes needs to be available for extraction. The search was limited to studies published in 1996 or later. Because of the advancements in POCUS in the last 25 years, we did not expect any relevant studies to have been conducted before 1996. No search limits were placed on the language of publication. Six authors selected the articles and extracted the data. Each step in the selection and data extraction process was done independently by two of these authors (Figure 1). The reviewers were not blinded to the authorship, journal, or year. Disagreements were resolved by consensus-based discussion, and when necessary, a third author adjudicated any disagreements. We extracted data regarding study design, study location, sample size, characteristics of participants, intervention, control group, reference standard, and outcome measures. Two authors independently assessed the quality of the studies with the Quadas-2 tool [24] for determining the risk of bias (Table 1).

**Table 1:** Quadas-2 tool for assessing the risk of bias. H: high risk of bias; L: low risk of bias; U: undetermined risk of bias *This refers to both included publications by Atkinson et al. [28,30]. A post-hoc analysis of the 2018 prospective study was published in 2019, dividing patient groups into cardiogenic or non-cardiogenic shock types [24].

Study	Patient selection	Index test	Reference standard	Flow and timing	Patient selection	Index test	Reference standard
Jones et al. [25]	L	L	L	L	L	L	L
Shokoohi et al. [26]	H	L	L	L	L	L	L
Sasmaz et al. [27]	U	L	H	U	L	L	H
Atkinson et al.* [28,30]	U	L	L	L	L	L	L
Javali et al. [29]	H	L	L	L	U	L	L

Outcome Measures and Data Analysis

A meta-analysis was not feasible because of the heterogeneity between the included studies. Therefore, study results were directly compared and critically appraised against the primary and secondary outcomes of the study.

Results

Search Results and Study Selection

The removal of duplicate studies resulted in 10,805 unique citations. After excluding 10,714 articles by screening the titles and abstracts, 91 articles were analyzed in more depth to assess their suitability. A further 85 articles that did not meet the inclusion criteria were therefore excluded (Appendices: Table 5). A flow diagram of the literature search is presented in Figure 1.

Study Characteristics

A total of six studies met the inclusion criteria [25-30]. The number of patients included varied from 100 to 270 patients per study, with a total of 852 patients in all studies together. Two studies were original randomized controlled trials (RCT) [25,28], and two studies had a prospective before-after design [26,27]. One study [30] was a post hoc analysis of a prospective trial [28], which is also included in this review. One study was a prospective explorative study [29]. All studies were published in English. Two studies were conducted in the USA [25,26], one study in both Canada and South Africa [28,30], one study in Turkey [27], and one study in India [29]. There was a moderate degree of variability in the quality of the included studies (Table 1). Three studies were judged to have a low risk of bias [25,28,30]. The three others were considered moderate to high-risk in one or more domains [26,27,29]. An overview of the study characteristics is presented in Table 2.

**Table 2:** Study characteristics. POCUS: point of care ultrasound, US: ultrasound, RCT: randomized controlled trial, USA: United States of America, ED: emergency department, Sx: Subxiphoid, PSLA: parasternal long axis, PSSA: parasternal short axis, A4C: apical 4 chamber, IVC: inferior vena cava, Aao: abdominal aorta, RUQ: right upper quadrant, LUQ: left upper quadrant, DVT: deep venous thrombosis, FOCUS: focused cardiac ultrasound, FAST: focused assessment with sonography in trauma, EP: emergency physician. *This study was a post-hoc analysis of the 2018 prospective study, dividing patient groups in cardiogenic or non-cardiogenic shock types.

Source	Design	Country	Setting	POCUS	US Machine	Operator
Jones et al. [25]	RCT, immediate vs delayed US	USA	ED (academic tertiary)	Sx, PSLA, A4C, IVC, Aao, RUQ, Pelvis	Shimadzu SDU-400	Treating EP and EP Residents
Shokoohi et al. [26]	Prospective "before-and-after"	USA	ED (academic tertiary)	Sx, PSLA, PSSA, A4C, Lungs (anterior and basolateral), IVC, Aao, Abdomen (FAST protocol)	Sonosite M-Turbo	EP not directly involved in patient care
Sasmaz et al. [27]	Prospective "before-and-after"	Turkey	ED (academic tertiary)	FOCUS, Lungs (anterolateral and base), IVC, Aao, RUQ, DVT	Esaote MyLab Class	Treating EP
Atkinson et al.* [28,30]	RCT	Canada, South Africa	ED (Canada: 3 large tertiary; South Africa: 1 large district, 1 large regional; 1 academic tertiary)	Sx, PSLA, PSSA, A4C, Lungs (base), IVC, Aao, RUQ, LUQ, Pelvis	Not specified	Treating EP
Javali et al. [29]	Prospective explorative	India	ED (academic tertiary)	Sx, PSLA, PSSA, A4C, Lungs, IVC, Aao, RUQ, LUQ, Pelvis, DVT	SonoSite M-TURBO	EP not directly involved in patient care

All studies took place in the ED [25-29]. Four of those were single-center studies, and Atkinson et al. [28,30] was a multicenter study. One study had a control group where no ultrasound was performed [28,30], and one study had a control group that received an ultrasound at a later stage after collecting the initial data [25]. The three other studies collected pre-and post-ultrasound data in the same patient group [26,27,29]. The mean age of the included patients varied from 52 to 63 years. The results of the included studies are summarized in Table 3.

**Table 3:** Outcomes. *This study was a post-hoc analysis of the 2018 prospective study, classifying patients as cardiogenic or non-cardiogenic shock types [30]. SD: standard deviation, yrs: years, IQR: interquartile range, POCUS: point of care ultrasound, ED: emergency department, sBP: systolic blood pressure, SI: shock index, CPR: cardiopulmonary resuscitation, MI: myocardial infarction, Defib.: defibrillation, ACLS: advanced cardiovascular life support, DNR: do not resuscitate, CI: confidence interval, Sx: subxiphoid, PSLA: parasternal long axis, PSSA, parasternal short axis, A4C: apical 4 chambers, IVC: inferior vena cava, RUQ: right upper quadrant, LUQ: left upper quadrant, Aao: abdominal aorta, DVT: deep venous thrombosis, FOCUS: focused cardiac ultrasound, FAST: focused assessment with sonography for trauma, NA: not available, Diff.: difference.

Study, year	N	Population	Age (yrs)	Intervention (POCUS)	Comparison (no POCUS)	Primary outcomes	Secondary outcomes
Jones et al. [25]	184	Non-trauma patients presenting to the ED, ≥18 years, sBP<100 mmHg, SI>1 exclusion: CPR, defib., ACLS drugs before enrolment, MI, obvious cause of shock, referral	56 (SD: 16)	Sx, PSLA, PSSA, A4C, Lungs, IVC, Aao, RUQ, LUQ, pelvis, DVT	No POCUS initially performed	POCUS vs no POCUS: diagnostic accuracy: 80% (95% CI: 70–87%) vs 50% (95% CI: 40–60%)	NA
Shokoohi et al. [26]	118	Non-trauma patients presenting to the ED, >18 years, sBP < 90 mmHg after 1 L fluid bolus; exclusion: obvious cause of shock, DNR	61.6 (95% CI: 58.7–64.5)	Sx, PSLA, PSSA, A4C, Thorax, IVC, Aao, abdomen (FAST protocol)	The same group before POCUS	Before POCUS vs after POCUS: definitive diagnosis (type of shock): 0.8% vs 12.7% (diff.: 11.9%; 95% CI, 5.6–18.1)	Change in treatment plan in 24.6% after POCUS (n=29; 95% CI, 16.7–32.5)
Sasmaz et al. [27]	180	Non-trauma patients presenting to the ED, ≥18 years, sBP < 100 mmHg or SI > 1; exclusion: CPR, pregnant, MI, obvious cause of shock	63.33 (SD: 18.1)	Sx, PSLA, PSSA, A4C, lungs (anterior and basolateral), IVC, Aao, abdomen (FAST protocol)	Same group before POCUS	Before POCUS vs after POCUS: diagnostic accuracy final diagnosis 60.6% vs 85.0%	Change in treatment plan in 50% (n=90), New treatment plan in 22.3% (n=40)
Atkinson et al. [28]	270	Non-trauma patients presenting to the ED, >19 years, sBP<100 mmHg, SI>1; exclusion: CPR, pregnant, MI, obvious cause of shock	POCUS: 56 (IQR 53.4–59.8) Control: 58.5 (IQR 56.2–62.1)	Sx, PSLA, PSSA, A4C, lungs (base), IVC, Aao, RUQ, LUQ, Pelvis	No POCUS performed	NA	POCUS vs No POCUS: median fluid volume administration after 4 h (mL, IQR): 1611 (1467–1833) vs 1676 (1402–1926) Inotrope usage rate (%): 12.9 vs 9.3; Diff. 3.6 (95% CI: −4.1 to 11.2)
Atkinson et al.* [30]	261	Non-trauma patients presenting to the ED, >19 years, sBP<100 mmHg, SI>1; exclusion: CPR, pregnant, MI, obvious cause of shock	POCUS: 56 (IQR 53.4–59.8) Control: 58.5 (IQR 56.2–62.1)	Sx, PSLA, A4C, IVC, Aao, RUQ, Pelvis	No POCUS performed	NA	POCUS vs No POCUS: mean fluid volume administration at ED discharge (mL, 95% CI) in cardiogenic shock: 744 (356–1131) vs 680 (28–1332); mean fluid volume administration at ED discharge (mL, 95% CI) in non-cardiogenic shock: 1763 (1520–2006) vs 1881 (1554–2209) Inotrope usage rate (%, 95% CI) in cardiogenic shock: 17.6 (−0.4 to 35.8%) vs 11.8 (−3.5 to 27.1%); inotrope usage rate (%, 95% CI) in non-cardiogenic shock: 12.4 (6.3–18.5%) vs 8.8 (3.6 to 13.9%)
Javali et al. [29]	100	>18 years, sBP < 90 mmHg, SI > 1, at least one sign or symptom of hypoperfusion; exclusion: referral, trauma, postural or asymptomatic hypotension	51.7 (SD 18.9)	FOCUS, lungs (anterolateral and base), IVC, Aao, RUQ, DVT	No POCUS was initially performed	POCUS vs no POCUS: diagnostic accuracy (type of shock): 89% vs 45%	NA

Analysis of Outcomes

**Figure 2:**
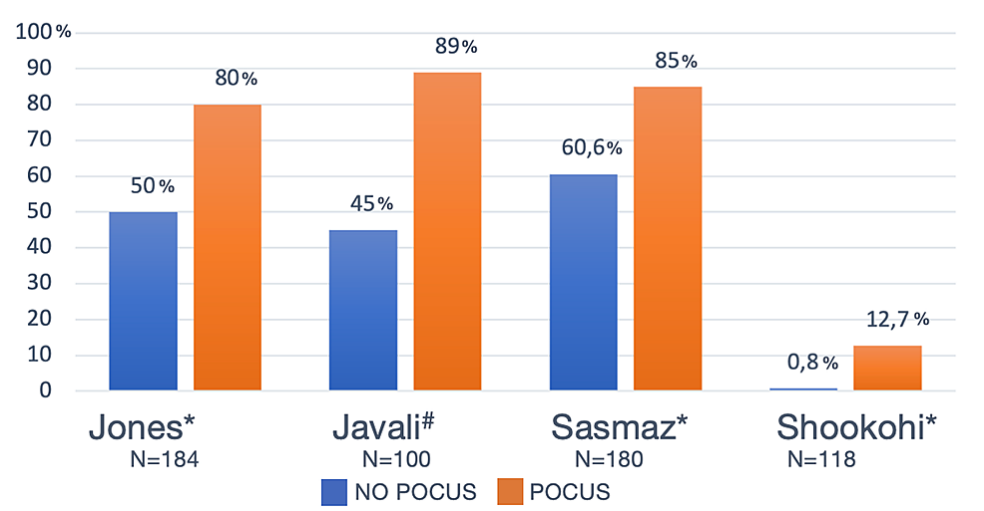
Diagnostic Accuracy No POCUS vs POCUS. Studies in figure from left to right: Jones [25], Javali [29], Sasmaz [27], Shookohi [26]. Three studies marked the difference in diagnostic accuracy between the two groups as significant (*) [25-27]. Javali [29] did not report significance but found a Cohen's kappa coefficient (#) of 0.89, correlating with an almost perfect agreement with the final diagnosis. The diagnostic accuracy was defined as the percentage of occurrence of the correct diagnosis with or without POCUS.

Diagnostic accuracy: Four out of six studies reported the diagnostic accuracy of a POCUS protocol for shock etiology in patients with undifferentiated shock in the ED and compared it to the diagnostic accuracy of a physician who did not use ultrasound (initially) as part of the workup [25-27,29]. Jones et al. and Sasmaz et al. looked at specific diagnoses [25,27], whereas Shokoohi et al. and Javali et al. examined diagnostic accuracy regarding different shock types [26,29]. An overview of the diagnostic accuracy in the No POCUS versus the POCUS group is presented in Figure 2. 

Jones et al. found that using POCUS in patients with undifferentiated shock, the diagnostic accuracy was 80%, compared to 50% in the control group that received no ultrasound at that point [25]. The 30% difference was significant (95% CI, 16-42%). The control group also received a POCUS exam after the first round of data collection, resulting in an increase in correct diagnoses from 50% to 78%. Similarly, Sasmaz et al. also found that diagnostic accuracy significantly increased from 61% before POCUS to 85% after POCUS [27].

Javali et al. reported that the accuracy in diagnosing the type of shock increased from 45% to 89% when adding POCUS by a trained emergency physician to the clinical information alone to make the diagnosis (an overall kappa correlation of 0.89) [29]. Shokoohi et al. [26] found a significant increase in patients with a definitive diagnosis for the type of shock from 0.8% before to 12.7% after POCUS was performed by an ultrasound-trained attending physician (Diff.: 11.9%; 95% CI, 5.6-18.1%). When they compared the final diagnosis with the leading POCUS diagnosis, it matched the discharge diagnosis in 86% of the cases (Cohen κ of 0.80; 95% CI, 0.73-0.88). 

Change of management: Four out of six studies reported on management changes [26-28,30]. Of these studies, however, only Atkinson et al. specified the difference in mean fluid volume administration and vasopressor use in the ED, both in the original study and its post-hoc analysis [28,30].

Fluid administration: Three out of six studies reported changes in ED fluid administration in patients after the use of POCUS [26,28,30]. One study mentioned changes in fluid regimens, yet did not report on statistical significance [26]. Atkinson et al. found no significant difference in the mean fluid volume administered during the first four hours between the POCUS and the standard care group [28]. A subgroup analysis that looked specifically at patients in cardiogenic shock also showed no significant difference in the mean amount of fluid administered between the POCUS and standard care groups [30].

Vasopressors: Three out of six studies reported on the use of vasoactive agents [26,28,30]. Atkinson et al. saw no significant difference in vasopressor usage, both in the original paper and in the post-hoc subgroup analysis [28,30]. Shokoohi et al. did report increased use of vasopressors after POCUS, ranging from 25 to 36%. This change, however, was not reported to be statistically significant [26].

Discussion

Evidence from the six available studies suggests that the use of POCUS in patients who presented to the ED with undifferentiated shock resulted in an increase in diagnostic accuracy of the shock type and final diagnosis, as well as a reduction in viable differential diagnoses and improved diagnostic confidence. However, we found no evidence of a change in fluid volume administration or use of vasopressors between the two groups.

In our review, diagnostic accuracy improved significantly from 45-60% to 80-89% when combined with clinical information. These results correlate well with those from other studies [31], including a recent systematic review and meta-analysis assessing the diagnostic accuracy of the RUSH exam for shock type in undifferentiated shock in the ED [11]. This study reported positive likelihood ratios (LR+) that ranged from 8.2 to 40.5, yielding clinically useful information, especially when ruling in a shock subtype. The positive likelihood ratios were highest for obstructive and lowest for mixed-etiology types of shock. A recent study published shortly after our search found that POCUS, when compared to standard examination, increased the accuracy of the cause of shock and altered the proposed treatment [32].

This high diagnostic accuracy was also expressed by the high concordance values between the diagnosis post POCUS and the final diagnosis in three of our studies, with overall Cohen’s kappa coefficients ranging between 0.80 and 0.89 [26,27,29]. These results were supported by previous studies where good to excellent concordance was found among the POCUS diagnosis, type of shock, and final diagnosis with inter-rater reliability Kappa coefficient values ranging between 0.70 and 0.97 [10,11,33-36].

In contrast to the other studies included in this review, accuracy numbers appeared low in Shokoohi et al., who had a strict protocol for diagnosing the type of shock where a diagnosis was termed definitive when a single diagnosis remained on the differential diagnosis sheet [26]. This appears to explain the lower accuracy finding of 0.8% before POCUS and 12.7% after POCUS introduction. However, when the initial leading POCUS diagnosis was compared to the final diagnosis, the diagnostic accuracy increased to 86%, which is comparable to the results found in the other studies [25,27,29].

Apart from the observed improvement in diagnostic accuracy, Jones et al. also found that the use of POCUS in patients with undifferentiated shock resulted in fewer viable diagnostic etiologies, with a median number of 4 in the POCUS group versus 9 in the control group (p<0.01) [25]. Furthermore, other studies found that POCUS led to higher physicians’ certainty regarding the diagnosis and cause of vital sign abnormalities in sepsis, chest pain, dyspnea, and symptomatic hypotension [26,33,37]. A similar increase in diagnostic confidence has also been reported in the ICU setting [38].

Therapeutic management changes were reported in four of the six selected studies [26-28,30]. Two studies reported treatment changes in 25% to 50% of cases [26,27]. However, these changes were not specified and may not have been significant or beneficial to the patient. Only Atkinson et al. investigated IV fluid volume administration and inotrope use in patients with undifferentiated shock in the ED and found no significant difference between the POCUS and standard care groups [28]. The same study’s post hoc analysis also failed to notice any treatment differences within both the cardiogenic and non-cardiogenic shock types when POCUS was compared against standard care [30]. These results are in contrast with findings from other ED studies that showed a change in treatment in patients that presented with sepsis [37] and hypotension [31]. In addition, many ICU studies also noticed significant treatment alterations brought on by the use of POCUS in patients that presented with shock [39], sepsis [38,40], and undifferentiated hypotension [41].

A plausible explanation for the lack of treatment changes in Atkinson et al. [28] could be the limited number of patients with POCUS-sensitive diagnoses. More than half of the patients included in this study were diagnosed with sepsis, which can lead to variable findings from hyper- to hypodynamic left ventricular function, variable inferior vena cava size and collapsibility, and findings such as ascites and pleural effusions. These findings make it difficult to make a correct diagnosis early. Other possible explanations for the lack of difference between the groups within this study were that comprehensive laboratory and advanced imaging resources were used in both groups, the high skill of emergency physicians and thus level of care, and that the definition of undifferentiated shock is still not accurate enough. An unclear definition could have led to the exclusion of patients before a final diagnosis was made. These patients could possibly have had the benefit of POCUS and could have contained POCUS-sensitive diagnoses. The hypothesis that POCUS-sensitive diagnoses occur rarely but can change treatment is supported by the findings of Shokoohi et al., who reported that there was a drastic change in management in only 5.1% of the cases in the population of ED patients with undifferentiated shock [42]. 

A recent study by Mosier et al. suggested that POCUS could lead to a delay in treatment and found higher mortality in the POCUS group [39]. However, this study has been reported to contain potential methodological weaknesses and, therefore, should be interpreted with caution when looking at the effect of POCUS in shock patients in the ED. In a letter to the editor, Amini et al. commented that the study had an unclear definition of POCUS, with inappropriate data inclusion and collection, and overstated conclusions. They reported that the study included educational studies that were not used for medical decision-making or related to interventions, thus introducing a bias [43]. In the studies included in this review, two studies reported on discordant diagnoses and potential harm. Both studies reported no indication of harm in the POCUS group compared to standard care, and no ultrasound findings were reported to lead to further unnecessary invasive procedures [25,28].

Clinical Implications and Future Perspectives

Since all studies were conducted in advanced care settings where other imaging modalities are widely available, the added value of POCUS in increasing diagnostic accuracy in some studies could be underestimated when compared to a medium- to low-resource setting. However, the same could be expected for treatment changes, but a sub-analysis comparing the South-African cohort with the Canadian cohort showed no difference between the POCUS and standard care group [28]. Further studies could potentially focus on diagnostic and therapeutic changes in medium- to low-resource settings. Another possible advantage is that POCUS can provide a more accurate diagnosis early and diminish the number of viable diagnoses. This could lead to less advanced imaging and examinations and, thereby, lower healthcare costs and time spent in the ED.

The most substantial added value of POCUS, therefore, seems to lie in its potential to increase both the diagnostic accuracy of the final diagnosis and shock type. In specific cases, POCUS seems to have the potential to shorten the time to a diagnosis, tailor and accelerate the workup and start of correct treatment, and prevent adverse outcomes. Future research on POCUS on patients in shock in advanced healthcare systems could focus on specific populations, patient- and setting tailored use of POCUS, and outcomes such as time-to-correct diagnosis, correct classification of shock type and diagnosis, and time to the next diagnostic examination. As a consequence, the time to appropriate treatment, treatment effects, and prevention of errors by using POCUS in high-risk decisions and patients could be influenced. Also, repetitive examinations during fluid and inotrope administration could positively guide treatment and outcomes in specific cases. Patient outcome benefits such as mortality and morbidity are of interest in future studies, and differences are more likely to be found in specific cases and high-risk settings, in contrast to expecting that one protocol would be useful for the entire population of undifferentiated shock patients. This is supported by reports of selected cases where it does seem likely that ultrasound can rapidly change diagnosis and treatment, lowering mortality and morbidity in shock patients, as described in a case series by Shokoohi et al. [42]. In a study by Gaspari et al., it was found that patients with pulseless electrical activity during cardiac arrest with organized activity, visualized on ultrasound, demonstrated an increased survival to hospital admission when started on adrenergic agents during resuscitation, compared to the group with disorganized activity [44]. Similar findings have been reported by Atkinson et al., who reported that patients with cardiac activity on POCUS had longer resuscitation times, were more likely to achieve ROSC, and had better survival to hospital discharge when compared to those without cardiac activity on POCUS [45]. Although these patients are at the far end of the shock spectrum, these findings support the hypothesis that ultrasound-guided therapeutic decisions might reduce mortality in patients in shock. However, looking at the undifferentiated shock in the ED, Atkinson et al. found no difference in the 30-day survival rate [28], nor in resuscitation outcome markers such as lactate, bicarbonate, Modified Early Warning Score (MEWS), or Shock Index (SI) [30].

Studying applications of POCUS within specific groups within the undifferentiated shock population can guide us to a tailored and time-effective approach for each scenario and care of each patient in undifferentiated shock. We, therefore, support an etiology-based prioritization of POCUS views as proposed in the IFEM consensus statement ShoC [22].

Limitations

Besides its strengths, this systematic review also has several limiting factors. We do not think that a selection or retrieval bias affected the results. Our study did not include specific populations such as children or pregnant patients and therefore may not accurately represent these patients. We did observe significant heterogeneity in reporting methods and a marked difference in the POCUS windows used between the included studies. Although there was a difference in POCUS windows, the included studies all included cardiac, IVC, aorta, and peritoneal views, and five out of six studies included thoracic views to assess for pneumothorax, pleural fluid, and pulmonary edema [26-30]. Due to the heterogeneity of the studies and varying definitions of diagnostic accuracy, we concluded that a meta-analysis would not add any value. Also, only two out of the six studies had sonographers who were not directly involved in patient care [26,29], and none of these provided quality assurances by reviewing the ultrasound images by a blinded assessor. The quality of reporting for the included studies was modest, with three of the six studies having a low risk of bias [25,28,30], while the other three studies had a moderate to high risk [26,27,29]. Moreover, two out of the six studies did not provide sample size calculations [26,27]. Therefore, the findings of this review might be influenced by the lack of power of the individual studies to detect a difference in the outcomes of interest. A publication bias could be a possible concern because studies, where no POCUS benefit was found, may not have been published.

## Conclusions

This systematic review demonstrates that POCUS improved the diagnostic accuracy of the underlying shock type in patients presenting with undifferentiated shock in the ED, compared to when clinical assessment without POCUS was used. Furthermore, POCUS use also improved the diagnostic accuracy of the underlying cause(s) of the shock type. POCUS use made no difference in intravenous fluid therapy or vasopressor management of patients presenting with undifferentiated shock. A subgroup analysis that looked specifically at patients in cardiogenic shock and non-cardiogenic shock also showed no significant difference in the mean amount of fluid administered between the POCUS and control groups. Since all studies were conducted in advanced care settings where other imaging modalities are widely available, the added value of POCUS could possibly be underestimated in a medium- to low-resource setting. These results should be interpreted within the scope of the limitations of the six studies included in the systematic review.

## Appendices

**Table 4:** Search strategy systematic review.

Date	Database	Search strategy	Number of references
May 5, 2019	PubMed (www.pubmed.gov)	(((shock[tiab] NOT ("shock wave"[tiab] OR "shock waves"[tiab])) OR circulatory failure*[tiab] OR circulatory collaps*[tiab] OR circulation collaps*[tiab] OR "collapsed circulation"[tiab] OR "collapse of circulation"[tiab] OR critical ill*[tiab] OR critically ill*[tiab] OR hypotens*[tiab] OR hypo-tens*[tiab] OR (low[tiab] AND (blood pressure*[tiab] OR bloodpressure*[tiab])) OR hemodynamically unstab*[tiab] OR "hemo-dynamically unstable"[tiab] OR haemodynamically unstab*[tiab] OR "haemo-dynamically unstable"[tiab] OR hemodynamic instab*[tiab] OR "hemo-dynamic instability"[tiab] OR "hemo-dynamic instabilities"[tiab] OR haemodynamic instab*[tiab] OR "haemo-dynamic instability"[tiab] OR "haemo-dynamic instabilities"[tiab] OR hemodynamic unstab*[tiab] OR "hemodynamic unstable"[tiab] OR hemodynamic unstable *[tiab] OR "hemo-dynamic unstable"[tiab] OR "hemo-dynamic unstability"[tiab] OR "hemo-dynamic unstabilities"[tiab] OR haemodynamic unstab*[tiab] OR "haemodynamic unstable"[tiab] OR "haemo-dynamic unstable"[tiab] OR "haemo-dynamic unstability"[tiab] OR "haemo-dynamic unstabilities"[tiab] OR hypovolem*[tiab] OR hypo-volem*[tiab] OR hypovolaem*[tiab] OR hypo-volaem*[tiab] OR septic*[tiab] OR sepsis*[tiab]) AND (ultraso*[tiab] OR ultra-so*[tiab] OR echo[tiab] OR echos[tiab] OR echo’s[tiab] OR echoc*[tiab] OR echo-c*[tiab] ORechog*[tiab] OR echo-g*[tiab] OR echoscop*[tiab] OR echo-scop*[tiab] OR echoso*[tiab] OR echo-so*[tiab] OR echotomo*[tiab] OR echo-tomo*[tiab] ORsonogra*[tiab] OR sono-gra*[tiab]) AND (emergenc*[tiab] OR emer-genc*[tiab] OR ed[tiab] OR eds[tiab] OR ed's[tiab] OR er[tiab] OR ers[tiab] OR er's[tiab] OR ccu[tiab] OR ccus[tiab] OR ccu’s[tiab] OR icu[tiab] OR icus[tiab] OR icu's[tiab] OR intensive*[tiab] OR itu[tiab] OR itus[tiab] OR itu's[tiab] OR critical*[tiab] OR high care*[tiab] OR highcare*[tiab] OR bedside*[tiab] OR bed-side*[tiab] OR focused*[tiab] OR rapid*[tiab] OR goal directed*[tiab] OR goaldirected*[tiab] OR guided*[tiab] OR echographyguided*[tiab] OR echography-guided*[tiab] OR echoguided*[tiab] OR echo-guided*[tiab] OR sonographyguided*[tiab] OR sonography-guided*[tiab] OR ultrasoundguided*[tiab] OR ultrasound-guided*[tiab] OR point of care*[tiab] OR points of care*[tiab] OR protocol*[tiab]) AND "1996/01/01"[PDAT]: "3000/12/31"[PDAT]) NOT (("Animals"[Mesh] NOT "Humans"[Mesh]) OR "Advertisements"[Publication Type] OR "Animation"[Publication Type] OR "Architectural Drawings"[Publication Type] OR "Biography"[Publication Type] OR "Book Illustrations"[Publication Type] OR "Bookplates"[Publication Type] OR "Charts"[Publication Type] OR "Comment"[Publication Type] OR "Editorial"[Publication Type] OR "Electronic Supplementary Materials"[Publication Type] OR "News"[Publication Type] OR "Patient Education Handout"[Publication Type] OR "Published Erratum"[Publication Type] OR "Retraction of Publication"[Publication Type] OR "Abbreviations"[Publication Type] OR "Academic Dissertations"[Publication Type] OR "Account Books"[Publication Type] OR "Addresses"[Publication Type] OR "Advertisements"[Publication Type] OR "Almanacs"[Publication Type] OR "Anecdotes"[Publication Type] OR "Animation"[Publication Type] OR "Annual Reports"[Publication Type] OR "Aphorisms and Proverbs"[Publication Type] OR "Architectural Drawings"[Publication Type] OR "Atlases"[Publication Type] OR "Bibliography"[Publication Type] OR "Biography"[Publication Type] OR "Book Reviews"[Publication Type] OR "Broadsides"[Publication Type] OR "Catalogs"[Publication Type] OR "Chronology"[Publication Type] OR "Collected Works"[Publication Type] OR "Collections"[Publication Type] OR "Comment"[Publication Type] OR "Congresses"[Publication Type] OR "Cookbooks"[Publication Type] OR "Diaries"[Publication Type] OR "Dictionary"[Publication Type] OR "Directory"[Publication Type] OR "Documentaries and Factual Films"[Publication Type] OR "Duplicate Publication"[Publication Type] OR "Editorial"[Publication Type] OR "Encyclopedias"[Publication Type] OR "Ephemera"[Publication Type] OR "Eulogies"[Publication Type] OR "Examination Questions"[Publication Type] OR "Exhibitions"[Publication Type] OR "Fictional Works"[Publication Type] OR "Forms"[Publication Type] OR "Formularies"[Publication Type] OR "Handbooks"[Publication Type] OR "Historical Article"[Publication Type] OR "Humor"[Publication Type] OR "Incunabula"[Publication Type] OR "Indexes"[Publication Type] OR "Instructional Films and Videos"[Publication Type] OR "Laboratory Manuals"[Publication Type] OR "Lecture Notes"[Publication Type] OR "Legal Cases"[Publication Type] OR "Legislation"[Publication Type] OR "Meeting Abstracts"[Publication Type] OR "Monograph"[Publication Type] OR "News"[Publication Type] OR "Newspaper Article"[Publication Type] OR "Nurses' Instruction"[Publication Type] OR "Outlines"[Publication Type] OR "Overall"[Publication Type] OR "Patents"[Publication Type] OR "Periodical Index"[Publication Type] OR "Periodicals"[Publication Type] OR "Pharmacopoeias"[Publication Type] OR "Photographs"[Publication Type] OR "Pictorial Works"[Publication Type] OR "Poetry"[Publication Type] OR "Popular Works"[Publication Type] OR "Postcards"[Publication Type] OR "Problems and Exercises"[Publication Type] OR "Programmed Instruction"[Publication Type] OR "Published Erratum"[Publication Type] OR "Retracted Publication"[Publication Type] OR "Retraction of Publication"[Publication Type] OR "Statistics"[Publication Type] OR "Tables"[Publication Type] OR "Technical Report"[Publication Type] OR "Unedited Footage"[Publication Type] OR "Union Lists"[Publication Type] OR "Unpublished Works"[Publication Type] OR "Video-Audio Media"[Publication Type] OR "Webcasts"[Publication Type] OR "Case Reports"[Publication Type] OR "Clinical Conference"[Publication Type] OR "Consensus Development Conference"[Publication Type] OR "Twin Study"[Publication Type])	4076
May 19, 2019	Embase, via OVID	'Advanced Search' Limits: -Publication year'1996 - current'. (((shock.ti,ab. NOT ("shock wave".ti,ab. OR "shock waves".ti,ab.)) OR circulatory failure*.ti,ab. OR circulatory collaps*.ti,ab. OR circulation collaps*.ti,ab. OR collapsed circulation*.ti,ab. OR collapse of circulation*.ti,ab. OR critical ill*.ti,ab. OR critically ill*.ti,ab. OR hypotens*.ti,ab. OR hypo-tens*.ti,ab. OR (low.ti,ab. AND (blood pressure*.ti,ab. OR bloodpressure*.ti,ab.)) OR hemodynamically unstab*.ti,ab. OR hemo-dynamically unstab*.ti,ab. OR haemodynamically unstab*.ti,ab. OR haemo-dynamically unstab*.ti,ab. OR hemodynamic instab*.ti,ab. OR hemo-dynamic instab*.ti,ab. OR haemodynamic instab*.ti,ab. OR haemo-dynamic instab*.ti,ab. OR hemodynamic unstab*.ti,ab. OR hemo-dynamic unstab*.ti,ab. OR haemodynamic unstab*.ti,ab. OR haemo-dynamic unstab*.ti,ab. OR hypovolem*.ti,ab. OR hypo-volem*.ti,ab. OR hypovolaem*.ti,ab. OR hypo-volaem*.ti,ab. OR septic*.ti,ab. OR sepsis*.ti,ab.) AND (ultraso*.ti,ab. ORultra-so*.ti,ab. OR echo.ti,ab. OR echos.ti,ab. OR echo's.ti,ab. ORechoc*.ti,ab. OR echo-ca*.ti,ab. ORechog*.ti,ab. OR echo-gr*.ti,ab. OR echo-gu*.ti,ab. OR echoscop*.ti,ab. OR echo-scop*.ti,ab. OR echoso*.ti,ab. OR echo-so*.ti,ab. OR echotomo*.ti,ab. OR echo-tomo*.ti,ab. OR sonogra*.ti,ab. OR sono-gra*.ti,ab.) AND (emergenc*.ti,ab. ORemer-genc*.ti,ab. OR ed.ti,ab. OR eds.ti,ab. OR ed's.ti,ab. OR er.ti,ab. OR ers.ti,ab. OR er's.ti,ab. OR ccu.ti,ab. OR ccus.ti,ab. OR ccu's.ti,ab. OR icu.ti,ab. OR icus.ti,ab. OR icu's.ti,ab. OR intensive*.ti,ab. OR itu.ti,ab. OR itus.ti,ab. OR itu's.ti,ab. OR critical*.ti,ab. OR high care*.ti,ab. OR highcare*.ti,ab. OR bedside*.ti,ab. OR bed-side*.ti,ab. OR focused*.ti,ab. OR rapid*.ti,ab. OR goal directed*.ti,ab. OR goaldirected*.ti,ab. OR guided*.ti,ab. OR echographyguided*.ti,ab. OR echography-guided*.ti,ab. OR echoguided*.ti,ab. OR echo-guided*.ti,ab. OR sonographyguided*.ti,ab. OR sonography-guided*.ti,ab. OR ultrasoundguided*.ti,ab. OR ultrasound-guided*.ti,ab. OR point of care*.ti,ab. OR points of care*.ti,ab. OR protocol*.ti,ab.)) NOT ((exp animal/ NOT exp human/) OR book.pt. OR book book.pt. OR "book review".pt. OR book series article.pt. OR book series book.pt. OR book series conference paper.pt. OR "book series conference review".pt. OR book series editorial.pt. OR book series erratum.pt. OR book series letter.pt. OR book series note.pt. OR "book series review".pt. OR book series short survey.pt. OR conference.pt. OR conference paper.pt. OR conference proceeding.pt. OR conference proceeding article.pt. OR conference proceeding conference paper.pt. OR conference proceeding editorial.pt. OR conference proceeding note.pt. OR "conference proceeding review".pt. OR editorial.pt. OR erratum.pt. OR note.pt.)	5865
May 24, 2019	Cochrane Library	Search Limits: -Tab 'search manager'. (((shock NOT ("shock wave" OR "shock waves")) OR circulatory NEXT failure* OR circulatory NEXT collaps* OR circulation NEXT collaps* OR collapsed NEXT circulation* OR collapse NEXT of NEXT circulation* OR critical NEXT ill* OR critically NEXT ill* OR hypotens* OR hypo-tens* OR (low AND (blood NEXTpressure* OR bloodpressure*)) OR hemodynamically NEXT unstab* OR hemo-dynamically NEXT unstab* OR haemodynamically NEXT unstab* OR haemo-dynamically NEXT unstab* OR hemodynamic NEXT instab* OR hemo-dynamic NEXT instab* OR haemodynamic NEXT instab* OR haemo-dynamic NEXTinstab* OR hemodynamic NEXT unstab* OR hemo-dynamic NEXT unstab* OR haemodynamic NEXT unstab* OR haemo-dynamic NEXT unstab* OR hypovolem* OR hypo-volem* OR hypovolaem* OR hypo-volaem* OR septic* OR sepsis*) AND (ultraso* OR ultra-so* OR echo OR echos OR echo’s OR echoc* OR echo-c* OR echog* OR echo-g* OR echoscop* OR echo-scop* OR echoso* OR echo-so* OR echotomo* OR echo-tomo* OR sonogra* OR sono-gra*) AND (emergenc* OR emer-genc* OR ed OR eds OR ed's OR er OR ers OR er's OR ccu OR ccus OR ccu’s OR icu OR icus OR icu's OR intensive* OR itu OR itus OR itu's OR critical* OR high NEXT care* OR highcare* OR bedside* OR bed-side* OR focused* OR rapid* OR goal NEXT directed* OR goaldirected* OR guided* OR echographyguided* OR echography-guided* OR echoguided* OR echo-guided* OR sonographyguided* OR sonography-guided* OR ultrasoundguided* OR ultrasound-guided* OR point NEXT of NEXT care* OR points NEXT of NEXT care* OR protocol*)):ti,ab,kw	1113 of which 1 editorial. This type of reference cannot be downloaded from the Cochrane Library.
September 14, 2015	PubMed (www.pubmed.gov)	(((shock[tiab] NOT ("shock wave"[tiab] OR "shock waves"[tiab])) OR circulatory failure*[tiab] OR circulatory collaps*[tiab] OR circulation collaps*[tiab] OR "collapsed circulation"[tiab] OR "collapse of circulation"[tiab] OR critical ill*[tiab] OR critically ill*[tiab] OR hypotens*[tiab] OR hypo-tens*[tiab] OR (low[tiab] AND (blood pressure*[tiab] OR bloodpressure*[tiab])) OR hemodynamically unstab*[tiab] OR "hemo-dynamically unstable"[tiab] OR haemodynamically unstab*[tiab] OR "haemo-dynamically unstable"[tiab] OR hemodynamic instab*[tiab] OR "hemo-dynamic instability"[tiab] OR "hemo-dynamic instabilities"[tiab] OR haemodynamic instab*[tiab] OR "haemo-dynamic instability"[tiab] OR "haemo-dynamic instabilities"[tiab] OR hemodynamic unstab*[tiab] OR "hemodynamic unstable"[tiab] OR hemodynamic unstable *[tiab] OR "hemo-dynamic unstable"[tiab] OR "hemo-dynamic unstability"[tiab] OR "hemo-dynamic unstabilities"[tiab] OR haemodynamic unstab*[tiab] OR "haemodynamic unstable"[tiab] OR "haemo-dynamic unstable"[tiab] OR "haemo-dynamic unstability"[tiab] OR "haemo-dynamic unstabilities"[tiab] OR hypovolem*[tiab] OR hypo-volem*[tiab] OR hypovolaem*[tiab] OR hypo-volaem*[tiab] OR septic*[tiab] OR sepsis*[tiab]) AND (ultraso*[tiab] OR ultra-so*[tiab] OR echo[tiab] OR echos[tiab] OR echo’s[tiab] OR echoc*[tiab] OR echo-c*[tiab] OR echog*[tiab] OR echo-g*[tiab] OR echoscop*[tiab] OR echo-scop*[tiab] OR echoso*[tiab] OR echo-so*[tiab] OR echotomo*[tiab] OR echo-tomo*[tiab] ORsonogra*[tiab] OR sono-gra*[tiab]) AND (emergenc*[tiab] OR emer-genc*[tiab] OR ed[tiab] OR eds[tiab] ORed's[tiab] OR er[tiab] OR ers[tiab] OR er's[tiab] OR ccu[tiab] OR ccus[tiab] OR ccu’s[tiab] OR icu[tiab] OR icus[tiab] OR icu's[tiab] OR intensive*[tiab] OR itu[tiab] OR itus[tiab] OR itu's[tiab] OR critical*[tiab] OR high care*[tiab] OR highcare*[tiab] OR bedside*[tiab] OR bed-side*[tiab] OR focused*[tiab] OR rapid*[tiab] OR goal directed*[tiab] OR goaldirected*[tiab] OR guided*[tiab] OR echographyguided*[tiab] OR echography-guided*[tiab] OR echoguided*[tiab] OR echo-guided*[tiab] OR sonographyguided*[tiab] OR sonography-guided*[tiab] OR ultrasoundguided*[tiab] OR ultrasound-guided*[tiab] OR point of care*[tiab] OR points of care*[tiab] OR protocol*[tiab]) AND "1996/01/01"[PDAT]: "3000/12/31"[PDAT]) NOT (("Animals"[Mesh] NOT "Humans"[Mesh]) OR "Advertisements"[Publication Type] OR "Animation"[Publication Type] OR "Architectural Drawings"[Publication Type] OR "Biography"[Publication Type] OR "Book Illustrations"[Publication Type] OR "Bookplates"[Publication Type] OR "Charts"[Publication Type] OR "Comment"[Publication Type] OR "Editorial"[Publication Type] OR "Electronic Supplementary Materials"[Publication Type] OR "News"[Publication Type] OR "Patient Education Handout"[Publication Type] OR "Published Erratum"[Publication Type] OR "Retraction of Publication"[Publication Type] OR "Abbreviations"[Publication Type] OR "Academic Dissertations"[Publication Type] OR "Account Books"[Publication Type] OR "Addresses"[Publication Type] OR "Advertisements"[Publication Type] OR "Almanacs"[Publication Type] OR "Anecdotes"[Publication Type] OR "Animation"[Publication Type] OR "Annual Reports"[Publication Type] OR "Aphorisms and Proverbs"[Publication Type] OR "Architectural Drawings"[Publication Type] OR "Atlases"[Publication Type] OR "Bibliography"[Publication Type] OR "Biography"[Publication Type] OR "Book Reviews"[Publication Type] OR "Broadsides"[Publication Type] OR "Catalogs"[Publication Type] OR "Chronology"[Publication Type] OR "Collected Works"[Publication Type] OR "Collections"[Publication Type] OR "Comment"[Publication Type] OR "Congresses"[Publication Type] OR "Cookbooks"[Publication Type] OR "Diaries"[Publication Type] OR "Dictionary"[Publication Type] OR "Directory"[Publication Type] OR "Documentaries and Factual Films"[Publication Type] OR "Duplicate Publication"[Publication Type] OR "Editorial"[Publication Type] OR "Encyclopedias"[Publication Type] OR "Ephemera"[Publication Type] OR "Eulogies"[Publication Type] OR "Examination Questions"[Publication Type] OR "Exhibitions"[Publication Type] OR "Fictional Works"[Publication Type] OR "Forms"[Publication Type] OR "Formularies"[Publication Type] OR "Handbooks"[Publication Type] OR "Historical Article"[Publication Type] OR "Humor"[Publication Type] OR "Incunabula"[Publication Type] OR "Indexes"[Publication Type] OR "Instructional Films and Videos"[Publication Type] OR "Laboratory Manuals"[Publication Type] OR "Lecture Notes"[Publication Type] OR "Legal Cases"[Publication Type] OR "Legislation"[Publication Type] OR "Meeting Abstracts"[Publication Type] OR "Monograph"[Publication Type] OR "News"[Publication Type] OR "Newspaper Article"[Publication Type] OR "Nurses' Instruction"[Publication Type] OR "Outlines"[Publication Type] OR "Overall"[Publication Type] OR "Patents"[Publication Type] OR "Periodical Index"[Publication Type] OR "Periodicals"[Publication Type] OR "Pharmacopoeias"[Publication Type] OR "Photographs"[Publication Type] OR "Pictorial Works"[Publication Type] OR "Poetry"[Publication Type] OR "Popular Works"[Publication Type] OR "Postcards"[Publication Type] OR "Problems and Exercises"[Publication Type] OR "Programmed Instruction"[Publication Type] OR "Published Erratum"[Publication Type] OR "Retracted Publication"[Publication Type] OR "Retraction of Publication"[Publication Type] OR "Statistics"[Publication Type] OR "Tables"[Publication Type] OR "Technical Report"[Publication Type] OR "Unedited Footage"[Publication Type] OR "Union Lists"[Publication Type] OR "Unpublished Works"[Publication Type] OR "Video-Audio Media"[Publication Type] OR "Webcasts"[Publication Type] OR "Case Reports"[Publication Type] OR "Clinical Conference"[Publication Type] OR "Consensus Development Conference"[Publication Type] OR "Twin Study"[Publication Type])	2865
September 14, 2015	Embase via OVID	Gezocht in: 'Advanced Search' Ingeperkt op: -'Embase'. -jaar van publicatie '1996 - current'. (((shock.ti,ab. NOT ("shock wave".ti,ab. OR "shock waves".ti,ab.)) OR circulatory failure*.ti,ab. OR circulatory collaps*.ti,ab. OR circulation collaps*.ti,ab. OR collapsed circulation*.ti,ab. OR collapse of circulation*.ti,ab. OR critical ill*.ti,ab. OR critically ill*.ti,ab. OR hypotens*.ti,ab. OR hypo-tens*.ti,ab. OR (low.ti,ab. AND (blood pressure*.ti,ab. OR bloodpressure*.ti,ab.)) OR hemodynamically unstab*.ti,ab. OR hemo-dynamically unstab*.ti,ab. OR haemodynamically unstab*.ti,ab. OR haemo-dynamically unstab*.ti,ab. OR hemodynamic instab*.ti,ab. OR hemo-dynamic instab*.ti,ab. OR haemodynamic instab*.ti,ab. OR haemo-dynamic instab*.ti,ab. OR hemodynamic unstab*.ti,ab. OR hemo-dynamic unstab*.ti,ab. OR haemodynamic unstab*.ti,ab. OR haemo-dynamic unstab*.ti,ab. OR hypovolem*.ti,ab. OR hypo-volem*.ti,ab. OR hypovolaem*.ti,ab. OR hypo-volaem*.ti,ab. OR septic*.ti,ab. OR sepsis*.ti,ab.) AND (ultraso*.ti,ab. OR ultra-so*.ti,ab. OR echo.ti,ab. OR echos.ti,ab. OR echo's.ti,ab. OR echoc*.ti,ab. OR echo-ca*.ti,ab. OR echog*.ti,ab. OR echo-gr*.ti,ab. OR echo-gu*.ti,ab. OR echoscop*.ti,ab. OR echo-scop*.ti,ab. OR echoso*.ti,ab. OR echo-so*.ti,ab. OR echotomo*.ti,ab. ORecho-tomo*.ti,ab. OR sonogra*.ti,ab. OR sono-gra*.ti,ab.) AND (emergenc*.ti,ab. OR emer-genc*.ti,ab. OR ed.ti,ab. OR eds.ti,ab. OR ed's.ti,ab. OR er.ti,ab. OR ers.ti,ab. OR er's.ti,ab. OR ccu.ti,ab. OR ccus.ti,ab. OR ccu's.ti,ab. OR icu.ti,ab. OR icus.ti,ab. OR icu's.ti,ab. OR intensive*.ti,ab. OR itu.ti,ab. OR itus.ti,ab. OR itu's.ti,ab. OR critical*.ti,ab. OR high care*.ti,ab. OR highcare*.ti,ab. OR bedside*.ti,ab. OR bed-side*.ti,ab. OR focused*.ti,ab. OR rapid*.ti,ab. OR goal directed*.ti,ab. OR goaldirected*.ti,ab. OR guided*.ti,ab. OR echographyguided*.ti,ab. OR echography-guided*.ti,ab. OR echoguided*.ti,ab. OR echo-guided*.ti,ab. OR sonographyguided*.ti,ab. OR sonography-guided*.ti,ab. OR ultrasoundguided*.ti,ab. OR ultrasound-guided*.ti,ab. OR point of care*.ti,ab. OR points of care*.ti,ab. OR protocol*.ti,ab.)) NOT ((exp animal/NOT exp human/) OR book.pt. OR book book.pt. OR "book review".pt. OR book series article.pt. OR book series book.pt. OR book series conference paper.pt. OR "book series conference review".pt. OR book series editorial.pt. OR book series erratum.pt. OR book series letter.pt. OR book series note.pt. OR "book series review".pt. OR book series short survey.pt. OR conference.pt. OR conference paper.pt. OR conference proceeding.pt. OR conference proceeding article.pt. OR conference proceeding conference paper.pt. OR conference proceeding editorial.pt. OR conference proceeding note.pt. OR "conference proceeding review".pt. OR editorial.pt. OR erratum.pt. OR note.pt.)	3479
September 14, 2015	Cochrane Library	Gezocht in: 'search manager'. Ingeperkt op: - (((shock NOT ("shock wave" OR "shock waves")) OR circulatory NEXT failure* OR circulatory NEXT collaps* ORcirculation NEXT collaps* OR collapsed NEXT circulation* OR collapse NEXT of NEXT circulation* OR critical NEXT ill* OR critically NEXT ill* OR hypotens* OR hypo-tens* OR (low AND (blood NEXT pressure* OR bloodpressure*)) OR hemodynamically NEXT unstab* OR hemo-dynamically NEXT unstab* OR haemodynamically NEXT unstab* OR haemo-dynamically NEXT unstab* OR hemodynamic NEXTinstab* OR hemo-dynamic NEXT instab* OR haemodynamic NEXT instab* OR haemo-dynamic NEXTinstab* OR hemodynamic NEXT unstab* OR hemo-dynamic NEXT unstab* OR haemodynamic NEXT unstab* OR haemo-dynamic NEXT unstab* OR hypovolem* OR hypo-volem* OR hypovolaem* OR hypo-volaem* OR septic* OR sepsis*) AND (ultraso* OR ultra-so* OR echo OR echos OR echo’s OR echoc* OR echo-c* OR echog* OR echo-g* OR echoscop* OR echo-scop* OR echoso* OR echo-so* OR echotomo* OR echo-tomo* OR sonogra* OR sono-gra*) AND (emergenc* OR emer-genc* OR ed OR eds OR ed's OR er OR ers OR er's OR ccu OR ccus OR ccu’s OR icu OR icus OR icu's OR intensive* OR itu OR itus OR itu's OR critical* OR high NEXT care* OR highcare* OR bedside* OR bed-side* OR focused* OR rapid* OR goal NEXT directed* OR goaldirected* OR guided* OR echographyguided* OR echography-guided* OR echoguided* OR echo-guided* OR sonographyguided* OR sonography-guided* OR ultrasoundguided* OR ultrasound-guided* OR point NEXT of NEXT care* OR points NEXT of NEXT care* OR protocol*)):ti,ab,kw	383

**Table 5:** Exclusion full text.

Reference	Population	Intervention	Comparison	Outcome	Inclusion/exclusion	Include	Exclude because	PICO	One group	Review	Meta-analysis	Article full text not found	No or limited data, ongoing trial	High risk of bias	Total
Search 2015															
1 Ahmed	yes	no	no	no	Exclude PICO1			1							1
2 Akilli	no	no	no	no	Exclude PICO1			1							1
3 Andrus	no	yes	no	review	Exclude Review					1					1
4 Arbo	yes	no	no	other	Exclude PICO3(not used as diagnostic tool to determine cause shock)			1							1
5 Arntfield	no	no	no	review	Exclude (no systematic review)					1					1
6 Bajwa	no	yes	yes	mortality	Exclude PICO4 not all patients in shock			1							1
7 Balik	no	no	no	no	Exclude PCIO 5			1							1
8 Becker	no	no	no	no	Exclude PICO 6			1							1
9 Beraud	yes	yes	yes	no	Exclude PICO 7			1							1
10 Boussuges	yes	yes	no	no	Exclude PICO 8			1							1
11 Breitkreuz	no				Exclude (prenohospital) PICO9			1							1
12 Carr	no	yes	yes	Volume status	Exclude PICO 10			1							1
13 Christiansen	no	yes	yes		Exclude PICO 11			1							1
14 Dark	no	yes	no	Treatment failure	Exclude, only one group (1)			1	1						1
15 Dipti	no	yes	no	Meta-analysis	Exclude						1				1
16 Ferrada, Anand	no	yes	no	yes	Exclude, only one group (2)			1	1						1
17 Ferrada, Evans	no	yes	yes	Mortality etc	Exclude			1							1
18 Ferrada, Murthi	no	yes	yes	yes	Exclude (only one group) (3)			1	1						1
19 Ferrada, Vanguri	no	yes	yes	Change of therapy	Exclude, only one group (4)			1	1						1
20 Gunst	yes	yes	yes	no	Exclude, PICO (12)			1							1
21 Haydar	no	yes	yes	no	Exclude			1							1
22 Holmes	no	yes	no	no	Exclude, one group (5)			1	1						1
23 Hutchings	no	yes	no	Change in treatment	Exclude, one group (6)			1	1						1
24 Jensen	no	yes	no	no	Exclude, PICO (13)			1							1
25 Jones, Craddock	yes	yes	no	yes	Exclude one group (7)			1	1						1
26 Jones [25]	yes	yes	yes	yes	Include	1									1
27 Josephs	yes	no	no	no	Exclude			1	1						1
28 Josephs	no	no	no	no	Exclude; review					1					1
29 Kabrhel	no	no	no	no	Exclude; PICOS not matched (14), see note			1							1
30 Kanji	no	yes	yes	no	Exclude, population ICU			1							1
31 Khouli	no	yes	yes	yes	Exclude, one group (9)			1	1						1
32 Lamia	no	yes	no	no	Exclude; PICOS not matched (15) see note			1							1
33 MacharenoDelgado	no	yes	yes	no	Exclude, one group (10)			1	1						1
34 Manno	no	yes	no	yes	Exclude, one group (11)			1	1						1
35 Marcelino	no	no	no	no	Exclude, one group (12), not in English			1	1						1
36 Massuratti	no	yes	no	no	Exclude, not two groups (13)			1	1						1
37 Matek	no	yes	no	no	EXCLUDE one group (14) no comparison group (both ultrasound and CT)			1	1						1
38 Mclean	no	yes	no	yes	Exclude, no comparison (15)			1	1						1
39 Moore	yes	yes	no	LV function	Exclude, no comparison (16)			1	1						1
40 Muller	no	no	no		Exclude, the US, only one group (17) (performed by echocardiographer)			1	1						1
41 Murthi	no	yes	yes	CI and LV function	Exclude, one group (18)			1	1						1
42 Orme	no	no			Exclude, no comparison group (19), US performed by cardiologists			1	1						1
43 Pulido	no	no	no		Exclude; PICO not matched (16)no clinical intervention – descriptive only.			1							1
44 Schefold	no	yes	yes	CVP, EVLW,	Exclude, one group (19)			1	1						1
45 Sefidbakht	yes	no	no	no	Exclude; outcomes/PICO not matched (16)			1							1
46 Schillcutt	no	no	no		Exclude, PICO not matched (17)			1							1
47 Tchorz	no	no	no		Exclude; no comparison group (20)			1	1						1
48 Toongyo	no	yes	no	yes	Exclude, one group only 21)			1	1						1
49 Verma	no	no		no	Exclude; only one group (22), full echo at ICU admission			1	1						1
50 vieillardnoBaron	no	no	yes	no	Exclude; PICOS not matched (18)			1							1
51 VieillardnoBaron	no	no	no	no	Exclude: Review of the topic					1					1
52 Volpicelli	yes	yes	no	no	Exclude, only one group (23)			1	1						1
53 Wang	?	yes	yes	yes	Exclude English no Chinese							1			1
54 Weekes	no	yes	no	no	Exclude; not meeting PICO (19), not clinically applied			1							1
55 Wherrett	no	yes/no	yes	no	Exclude; Trauma not shock (population), one group (24)			1	1						1
56 Wu	Chinese article		no		Exclude; one group (25) (read from English abstract), not English			1	1						1
57 Yanagawa	yes	yes	yes	yes	Exclude, one group (26)			1	1						1
58 Zengin	yes	yes	yes	yes	Exclude, one group (27)			1	1						1
Atkinson (preliminary data provided by the author in 2015) Included in 2019 search															0
Bagheri	yes	yes	no	yes	One group			1	1						1
Ghane	yes	yes	no	yes	One group			1	1						1
Total 2015						1	0	53	30	4	1	1	5		65
New search 2019															
101. Dinc. 2015 Hong Kong Journal of Emergency Medicine	yes	yes	yes	yes	Exclude			1	1						1
102. Cortellaro 2017 Intern Emerg Med	no				Exclude			1							1
103 (already excluded in previous selection = study number 6 Bajwa 2012)					Exclude										0
104. Ahn 2017 PLoS One	yes	yes	no	no	Exclude			1	1						1
105. Bennet 2018 Cardiovasc Ultrasound	No				Exclude			1							1
106. Elbaih 2018 Chin J Traumatol	yes	yes	no		Exclude			1	1						1
107. Feng 2018 Intensive Care Med	No				Exclude			1							1
108. Bernierno Jean 2017 Journal of Intensive care medicine	No		No		Exclude			1	1						1
109. Baston 2018 American Journal of respiratory and critical care	No				Exclude			1							1
110. Atkinson 2018 Annals of Emergency Medicine [28]	Yes	Yes	Yes	Yes	Include	1									1
111. Guerin 2016 Clin Chest Med	Yes		No		Exclude			1	1						1
112. Hall 2017 J Intensive Care Soc	Yes		No		Exclude			1	1						1
113. Keikha 2018 Bull Emerg Trauma	No	No		No	Exclude						1				1
114. Milne 2013 Canadian journal of emergency medicine	Poster of Atkinson preliminary results				Exclude										0
115no116no117no118 NCT(4x) (non-published research? → screen other titles and email author if research is published yet) @ Kris	No				Exclude							1			1
116 NCT	No				Exclude							1			1
117 NCT	No				Exclude							1			1
118 NCT	No				Exclude							1			1
119. Peach 2017 Canadian Journal of Emergency Medicine	Summary abstract of atkinson ShocnoED study				Exclude										0
120. Sekiguchi 2017 J Anesth (included by JF, not Nadim)	yes	yes	yes	yes	Exclude			1							1
121. Tascini 2017 Internal and Emergency Medicine (included by JF, not Nadim)	No				Exclude								1		1
122. Rahul Kumar 2019 J Emerg Trauma Shock	yes	yes	no		Exclude			1	1						1
123. Sasmaz 2017 Emergency Medicine [27]	yes	yes	yes	yes	Include	1									1
124. Shokoohi 2015 Critic Care Med [26]	yes	yes	yes	yes	Include	1									1
125. Shokoohi 2017 American Journal of Emergency Medicine			no	no	Exclude			1	1						1
126. Taylor 2017 Canadian journal of emergency medicine	Abstract of SHOCnoED Study Atkinson				Exclude										0
Total 2019						3	0	13	8	0	1	4	1	1	22
Search 2020															
201. Mosier	yes	no	yes	no	Exclude			1							1
202. Javali [29]	yes	yes	yes	yes	Include	1									1
203. Atkinson Resuscitation markers	yes	yes	yes	no	Exclude			1							1
204. Atkinson Shock type [30]	yes	yes	yes	yes	Include	1									1
Total 2020						2		2	0	0	0	0	0	0	4
Total references 2015, 2019 and 2020 selection full text						6	0	68	38	4	2	5	6		91
CLIN GOV SEARCH search															0
Search 2015															
Atkinson	yes	yes	yes	yes	2018 study included with final results (2015 correspondence only yielded preliminary results)	0									0
5 Other articles selected for request information					Exclude, no data acquired								5		0
New search 12 April 2020					Exclude										0
Critical care ultrasound oriented shock treatment in ICU					Exclude		No data available after contacting trial contact								0
Focus cardiac ultrasound in patients with shock					Exclude		No data available after contacting trial contact								0
African resuscitation ultrasound in critically no ill adults					Exclude		No data available after contacting trial contact								0
The use of a point of no care thoracic ultrasound protocol for hospital medical emergency teams (METUS)					Exclude		No data available after contacting trial contact								0
RHAPSody: diagnostic utility of RUSH following ROSC					Exclude		No data available after contacting trial contact								0
Total CLIN GOV search															0
